# Pathobiological Mechanisms of Endothelial Dysfunction Induced by *tert*-Butyl Hydroperoxide via Apoptosis, Necrosis and Senescence in a Rat Model

**DOI:** 10.7150/ijms.40255

**Published:** 2020-02-04

**Authors:** Yueh-Chiao Yeh, Tsun-Jui Liu, Hui-Chin Lai

**Affiliations:** 1 Department of Natural Biotechnology, Nanhua University, Chiayi, Taiwan; 2 Cardiovascular Center, Department of Anesthesiology and Department of Internal Medicine, Taichung Veterans General Hospital, Taichung, Taiwan; 3 Department of Medicine, National Yang-Ming University, School of Medicine, Taipei, Taiwan

**Keywords:** apoptosis, cell cycle, p53, rat aortic endothelial cells, telomerase activity, *tert*-butyl peroxide (*t-*BHP)

## Abstract

**Background**: Endothelial dysfunction is one of the underlying causes for vascular diseases. *tert-Butyl* hydroperoxide (*t*-BHP), a short-chain lipid hydroperoxide analog, has been reported to cause adverse effects in different systems. However, the adverse actions of *t*-BHP on inducing endothelial dysfunction are unclear and remain under investigation. Aim of the present study was to identify the pathobiological mechanisms of *t*-BHP in rat aortic endothelial cells and thoracic aorta.

**Methods**: Primary cultured cells were treated with vehicle or *t*-BHP (50, 100, 250, 500, and 1,000 μM). Cells were harvested and specific analyses regarding cellular apoptosis, necrosis, and senescence were conducted. Additionally, *t*-BHP (0.1, 0.2, and 0.4 mmol/kg body weight) or vehicle were administered to male rats (the young group at 6 weeks of age and the mature adult group at 24 weeks of age) daily through intraperitoneal injections. At 10 days after the first drug treatment apoptotic endothelial toxicity was evaluated by biochemical, histological, and immunofluorescent staining analyses.

**Results**: Dose-dependent effects of *t*-BHP were observed for the reduction of cell viability, deterioration of cell toxicity, initiation of cell cycle arrest, and triggering of apoptosis and necrosis. Moreover, increase of cells stained positive for senescence-associated beta-galactosidase (SA-β-Gal), amelioration of telomerase activity, and precipitations of necrotic, cell cycle, and apoptotic signaling regulatory proteins were also found in the *in vitro* model. In the *in vivo* study, results indicated that *t*-BHP at higher doses enlarged the intima-medial thickness of descending aorta in the mature adult group, but led to aortic narrowing in the young group. Increased injuries were observed by upregulating endothelial apoptosis- and senescence-positive staining, along with caspase-3 activity and down-regulating telomerase activity.

**Conclusion**: These results confirmed that *t*-BHP impaired aortic endothelial cell survival at least partially by the activation of p53-mediated signaling pathways, inhibition of cell cycle regulatory proteins, and initiation of cellular senescence-related signaling pathways. In conclusion, *t*-BHP was found to be a major trigger for impairing aortic endothelial cell survival and deteriorating vascular dysfunction in experimental practice.

## Introduction

Aortic endothelial cells (ECs) are well known for their abilities to maintain homeostasis and hemostasis in the human cardiovascular system. Increased evidence suggested that endothelial dysfunction is involved in the initiation and progression of many cardiovascular diseases (CVDs) 1-3. The endothelial cells, which form a single continuous layer of blood vessels, are a distinctive target for cytotoxic or cytostatic effects induced by systemic oxidative stress and inflammatory imbalance 4-6. Previous reports demonstrated that dietary patterns and age are key factors strongly correlated with endothelial dysfunction to influence the early onset of coronary artery disease. In particular, the use of additives in processed foods has strong impacts on vascular endothelial functions 7-9.

*tert*-Butyl hydroperoxide (*t*-BHP), one of the pro-oxidant compounds used as additive in frozen seafood, lubricants, bleaching and disinfectant, is a free radical initiator that generates epoxidation of alpha-olefins with various choices of transition metal catalysts 10-11. It can trigger peroxyl and alkoxyl radicals and produce highly OH-radical-type reactive oxygen species (ROS) to disrupt cellular membranes, and damage cells by reacting with DNA, proteins, and lipids 12-14. There are growing reports also implicating *t*-BHP in the elicitation of acute and chronic toxicities in hepatocytes by deteriorating cell viability and proliferation, inducing oxidative stress, increasing serum levels of alanine aminotranferease (ALT) and aspartate aminotransferase (AST), upregulating expressions of apoptotic-related proteins, and activating capase-3 signaling pathway 10,12,15-17. Furthermore, it has been found to induce cellular injuries in rat astroglial cells 18, human trophoblast-derived cells 19, human corneal endothelial cells 20, mouse brain endothelial cells 21, mouse brain tissues 22, and rat testes 23. Previous studies also indicated that *t*-BHP trigger vasoconstriction in isolated rat thoracic aorta 24 and enhance contractile responses in aortic strips of diabetic rats 25, which might restrict or reduce blood flow and eventually increase the risk of morbidity and mortality.

Stress-induced premature senescence (SIPS) of vascular endothelial cells is implicated in destroying the production of vasoconstrictors and vasodilators, and triggering the disturbance of inflammatory processes. This dysfunctional endothelium in turn exacerbates the risk of various cardiovascular diseases 26. Particularly, it has been indicated that *t*-BHP is an inducer in modeling premature senescence, enhancing the rate of telomere shortening, inducing senescence-associated beta-galactosidase (SA-β-Gal) activity, and changing growth responses in WI-38 fibroblast cells 27 as well as lens epithelial cells 28. Telomerase activity is associated with resistance to apoptosis, and oxidative stress-induced DNA damage. Within all factors, p53 is the key determinant to trigger apoptosis and cellular senescence in response to various stressors, and p21 also can enforce the initial cell cycle arrest and negatively regulate p53-mediated apoptosis 29-30. Nuclear Factor-kappa B (NF-kB) is also considered as another component of vascular endothelial dysfunction through inflammatory imbalance 6. The activation of NF-kB is indicated to upregulate heat shock protein 60 (HSP60) expression in cardiomyocytes, stimulate the immune response, and further lead to mitochondrial apoptosis events 31. With these aforementioned observations, it remains to be seen whether this endothelial dysfunction could interrelate with *t*-BHP initiated apoptosis, necrosis-associated inflammatory responses, and premature senescence. Accordingly, this study addressed the possible dose-response effects of *t*-BHP in rat vascular endothelium on cell viability, apoptosis, cell cycle progression, cellular senescence, and necrosis mediated signaling pathways in rats with *in vivo* and *in vitro* experimental models to provide a practical approach for evaluating the adverse reactions to food additives in the human system.

## Materials and Methods

### Chemicals and reagents

3-(4,5-dimethylthiazol-2-yl)-2,5-diphenyl tetrazolium bromide (MTT), *tert*-butyl hydroperoxide (*t*-BHP), and antibody against β-actin (Actin) were obtained from Sigma (St. Louis, MO, USA). Dulbecco's modified Eagle's medium (DMEM) and fetal bovine serum (FBS) were purchased from GIBCO (New York, NY, USA). Propidium iodide (PI) was purchased from Molecular Probe (Eugene, OR, USA). Antibody against p53 was purchased from Lab Vision Corp. (Fremont, CA, USA). HRP-conjugated secondary antibodies to mouse, rabbit, and goat immunoglobulins were purchased from Invitrogen (Carlsbad, CA, USA). All other antibodies were from Santa Cruz Biotechnology (Santa Cruz, CA, USA), and all other chemicals and reagents used were of the highest commercial grades available.

### Animals and *in vivo* pharmacological treatment

Male Sprague-Dawley rats weighing 150-180 g (6 weeks of age as the young group) or 300-350 g (24 weeks of age as the mature adult group) 8,32 were purchased from BioLASCO Technology (Taipei, Taiwan). They were maintained in a controlled environment at room temperature with a 12-h dark/ light cycle and acclimatized for at least one week prior to use. For dose response experiments, a total of 48 rats for the young group and 24 rats for the mature adult group were randomly assigned to four groups and received the intraperitoneal (i.p.) administration of *t*-BHP at doses of 0.1, 0.2, and 0.4 mmol/kg body weight (BW) in 400 μl saline every day, for 10 consecutive injections 8,33. The cumulative doses were equivalent to 45, 90, or 180 mg for a 70 kg human, just above the threshold at which *t*-BHP induces cellular toxicity is expected to occur clinically 34. Control rats (Cont) were given the equivalent volumes of normal saline (the solvent for *t*-BHP) daily i.p. injections. All animals were euthanized 10 days after the first injection normal saline or* t*-BHP. The thoracic aorta were isolated for morphological and biomedical studies. The body weight gains were measured and compared with the Cont group. This investigation conformed to the *Guide for the Care and Use of Laboratory Animals* published by the US National Institute of Health (NIH Publication No. 85-23, revised 1996). The experimental procedures were approved by the Institutional Animal Care and Use Committee at Taichung Veterans General Hospital, Taiwan (No. La-98679, La-98680, and La-98681).

### Isolation of rat aortic endothelial cell and in vitro experimental protocol

Isolation of rat aortic endothelial cells (ECs) from primary explants was prepared from male Sprague-Dawley rats (4 weeks of age) as previously reported 35. Pure endothelial cells were maintained with 10 % FBS/DMEM at 37°C in an incubator with a humidified atmosphere of 5 % CO_2_. The confluent cell at passage numbers 3-6 exhibited a typical “cobblestone” growth pattern 35, which identified with the endothelium-specific antibody, von Willebrand Factor (vWF) 36 were used for the experiments. A density of 4 x10^5^ cells/mL seeded into 10-cm plates were treated with vehicle (normal saline) or *t*-BHP (50, 100, 250, 400, or 1000 μM) in 2 % FBS/DMEM for 15 min, 30 min, 1 hour, 2 h, or 24 h according to previous studies 10,18-19,35,37.

Changes of cell morphologies were observed under the CKX41 inverted phase-contrast light microscope (Olympus, Japan) and the digital images were captured by Spot CCD Camera which driven by Advanced Spot RT Software version 3.3. The cell viability in response to *t*-BHP treatment was assessed by MTT assays as describe by Yeh et al. 38. The optical density (O.D.) values reflected the MTT reductase activities and hence the amounts of viable cells. Results were expressed with respect the control. At least three experiments were conducted for each treatment, using the mean value for data analyses.

### Flow cytometric analysis by Propidium Iodide staining

Endothelial cells (ECs) treated with indicated concentrations of *t*-BHP for 24 hours were trypsinized, harvested, wash, and then resuspended in PBS. Afterwards, the cells were incubated with Ribonuclease A (0.1 mg/mL) and PI solution (50 μg/mL) in the dark for 30 min at 37^o^C. The stained ECs were filtered through a 40 mm nylon mesh and then differentiated using BD Biosciences FACScan system with CellQuest^TM^ Pro software (BD Biosciences, San Jose, CA, USA). The fluorescence emission of the PI-stained cells excited by a 488 nm argon ion laser was measured at a wavelength of 610 nm in a FACSVantage flow cytometer. The proportion of apoptotic cells in each sample was estimated from the subG1 peak in the DNA histogram. At least 10,000 events were used in calculations for each sample. Experiments were repeated a minimum of three times, and the average of subG1 peak ratios was regarded as the apoptotic index of the sample.

### Apoptosis and necrosis assays using fluorescence-conjugated Annexin-V (Annexin-V-FITC)/Propidium iodide staining

To analyze the endothelial cells (ECs) for apoptosis and necrosis, cells treated with or without *t*-BHP for 24 hours were trypsinized, pelleted, and resuspended in 1 x binding buffer containing both Annexin-V-FITC antibody and PI according to the manufacturer's indicated protocol. The samples were then analyzed by flow cytometry and the fluorescence emission of the FITC-stained cells excited by a 490 nm argon ion laser was measured at a wavelength of 525 nm in a FACSVantage flow cytometer. ECs were gated by forward and side scatter measurements. At least 10,000 events were used in calculations for each sample and the proportion of early apoptosis, late apoptosis, necrotic/dead, and viable cells, respectively.

### *In-situ* detection of apoptosis in endothelial cells and thoracic aorta

Apoptosis or programmed cell death was double checked by TdT-mediated dUTP-biotin nick-end labeling (TUNEL) assay using an In Situ Cell Death Detection Kit, Fluorescein (Roche, Basel, Switzerland). Tissue cryo-sections of rat aorta (10 μm in thickness) and endothelial cells were fixed in 4 % paraformaldehyde, digested with proteinase K (20 μg/ml), and treated with equilibrium buffer. The sections from each specimen and cells were counterstained with 4',6-diamidino-2-phenylindole (DAPI) and observed under fluorescence microscopy (Leica, DMR, Bensheim, Germany). Digital images were captured with a Spot CCD Camera driven by Advanced Spot RT Software version 3.3 (Diagnostic Instruments, Inc., Sterling Heights, MI, USA) to determine the proportion of cells positively stained by TUNEL.

### Determination of Caspase-3 activity in thoracic aorta and endothelial cells

Activities of caspase-3 in endothelial cells (ECs) and aortic tissues were estimated by their cleavage of the colorimetric substrate (Z-DEVD-R110) provided in the EnzChek® Caspase-3 Assay Kit System (Molecular Probes, Eugene, OR, USA). Briefly, fresh aorta samples frozen in nitrogen liquid or pelleted endothelial cells (about 5 x 10^6^) centrifuged at 450 x g for 10 min, were washed with ice-cold PBS, and resuspended in 50 μL of 1 X Cell Lysis Buffer. The 50 μl supernatants from each sample were transferred to individual microplate wells, with 50 μL of the 1 X Cell Lysis Buffer and 50 μL of the 2 X substrate working solution were added to each well and incubated at room temperature for 30 min. The fluorescence was measured (excitation/emission 496/520 nm) with fluorescence plate reader (Fluoroskan Ascent, Labsystems) and it represented the caspase-3 activity of this sample. Caspase-3 activity of endothelial cells was further evaluated by flow cytometry using a Casp-GLOW RED-Active Caspase-3 Staining Kit (BioVision, Mountain View, CA, USA) by flow cytometry using the FL-2 channel.

### Immunoblotting analysis

To detect cellular response to the stimulation of *t*-BHP in aspects of apoptotic signaling mediators (Bax, Bcl-2, and cytochrome *c*), inflammatory and necrotic regulators (HSP60 and NF-kB), and cell cycle regulated proteins (p53, p21) with immunoblotting, aortic tissues or pelleted cells were lysed on ice with a lysis buffer and were centrifuged at 10,000 x g at 4 ^o^C for 10 min. The supernatant was acquired and the protein concentration was determined. For the analysis of cytochrome *c* (cyto *c*), mitochondrial and cytosolic fractions were prepared as described previously 38. The density of each protein band was scanned using ScienceLab 2001 ImageGauge 4.0 Software (Fujifilm, Tokyo, Japan) and compared between groups by densitometry (Actin as a reference).

### Immunofluorescent microscopy

Endothelial cells (ECs) grown on glass cover slides were fixed with 4% paraformaldehyde/PBS for 30 min and blocking for nonspecific binding. Cells were then incubated with primary antibody at dilutions of 1:100 for 18 h at 4 ^o^C, washed twice in PBS/ 0.05% Triton X-100 solution, and reacted with a fluorescein-conjugated secondary antibody for 1 h at room temperature. Nuclei were counter-stained with DAPI containing mounting medium, and cells were then examined with a fluorescence microscope (Leica DMR, Bensheim, Germany) at a magnification of 400 X to determine contents.

### Senescence-associated β-galactosidase staining in endothelial cells and aortic tissues

Senescence-associated β-galactosidase (SA-β-Gal) reflects an increase in lysosomal mass during replicative aging and is a good marker of senescence 30. Briefly, cryo-sections of aortic sections (10 μm in thickness) and/or endothelial cells were washed in PBS, fixed for 3-5 mins in Fixative solution, washed, and incubated at 37 ^o^C (no CO_2_) with fresh SA-β-Gal stain solution. Staining was observed under a light microscope, and then the corresponding digital images were captured for later analysis by a Spot CCD Camera driven by Advanced Spot RT Software version 3.3 (Diagnostic Instruments Inc., MI, USA). The total numbers and the positive stained endothelial cells were assessed under microscope at 200 X magnification.

### Telomerase activity

Telomerase activity was detected by PCR-based Telomeric Repeat Amplification Protocol (TRAP) with a TRAPeze® XL telomerase detection kit (Millipore, Billerica, MA, USA), according to the manufacturer's instructions. The fluorescence was measured (excitation/emission 496/520 nm) with a fluorescence plate reader (Fluoroskan Ascent, Labsystems) and it represented the telomerase activity of this sample.

### Statistical analysis

All data are expressed as the mean ± standard error of the mean (S.E.M.). All experiments were repeated at least 3 times (≥ 3 replicates) on each specimen and there were 3 specimens from each group. The results of all replicates from each specimen were averaged, and the mean of averaged values from all specimens of a single group was regarded as the corresponding value of the whole group. Statistical analyses were performed using one-way ANOVA followed by Dunnett's *post hoc* analysis. The results were considered statistically significant if the p value was less than 0.05.

## Results

### Effects of *t*-BHP on body weight and morphometric parameters of aorta in experiment rats

All animals from each group were alive and their physical activities were near to normal during and/or after treatment. However, the dose-response effects of *t*-BHP administration on body weight were observed. During the initial day of treatment, there was no significant difference in body weight in all rats from both the young and mature adult groups. At the end of experiments, the body weight gains were significantly less in rats treated with higher dose of *t*-BHP (0.2 mmol/kg) (p=0.029 vs. Cont) in the young group and with the highest test dose of *t*-BHP (0.4 mmol/kg) (p= 0.004 vs. Cont) in the mature adult group (Table 1). The intima-medial thickness (IMT) of aortic rings has been reported as a good predictor of the risk of cardiovascular events 5. Our data showed that this index were notably decreased by higher doses of *t*-BHP in the young group. The aortic rings displayed normal endothelial and vascular morphologies in both control groups, while enlarged IMT of descending aorta were found in the mature adult group.

### Effects of t-BHP on endothelial cell viability, cell morphology, and cell cycling

**Figure 1:**
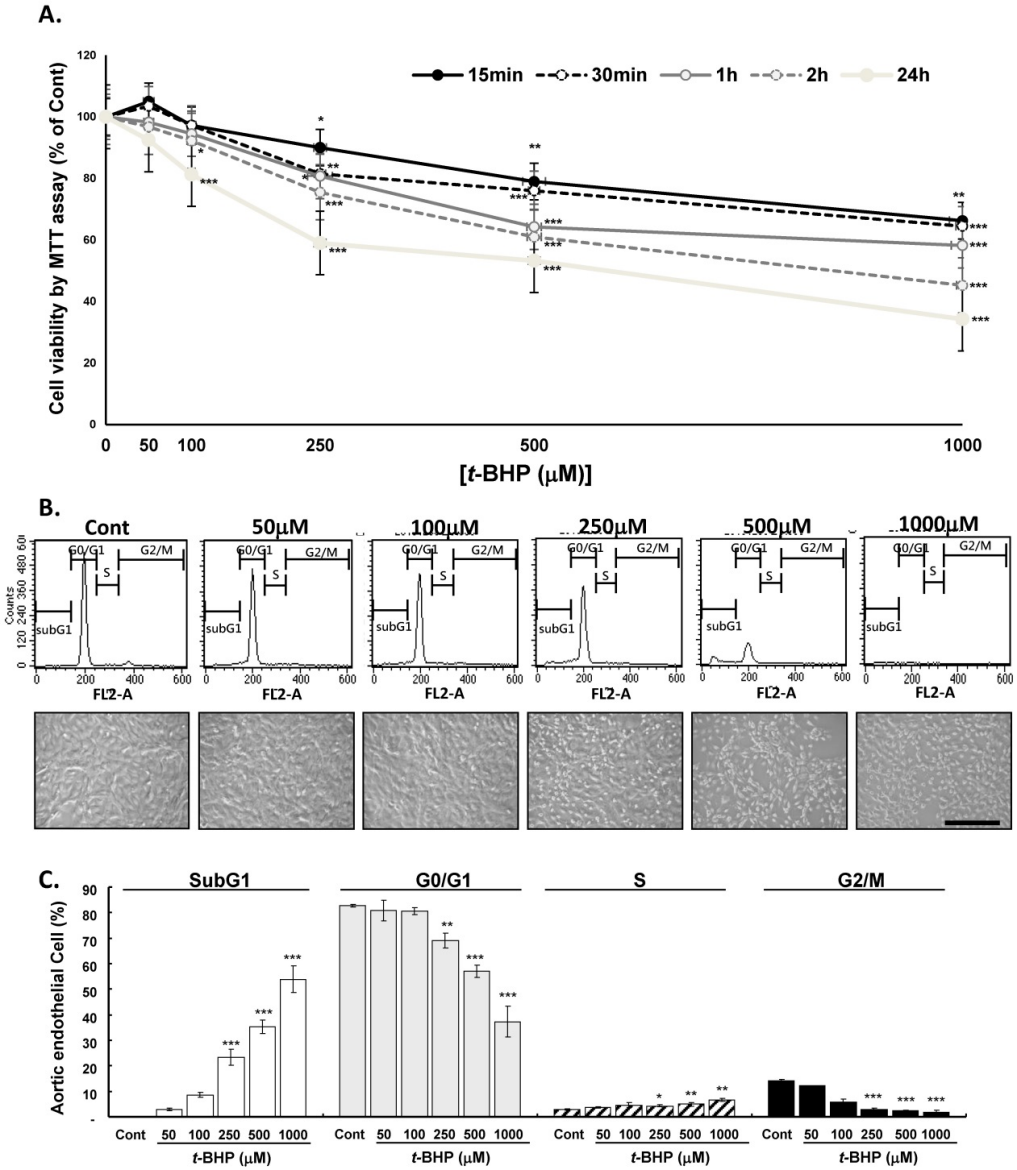
** Dose- and time- dependent effects of *tert*-butyl hydroperoxide (*t*-BHP) induced cytotoxicity in rat aortic endothelial cells (ECs).** The MTT assay was employed to assess the cell viability after *t*-BHP administration. **(A)** For dose-dependence, *t*-BHP was given at a concentration of 50, 100, 250, 500 and 1000 μM. The endothelial cells (ECs) treated with vehicle were as the Cont. For time-dependence, *t*-BHP was given for 15 min, 30 min, 1 hour, and 24 hours respectively to induce cell damage. Data were collected at least from three independent experiments and represented as mean ± S.E.M. **(B)** Representative DNA distribution histogram of ECs by flow cytometric analysis. The ECs were treated with indicated concentrations of *t*-BHP for 2 hours and then harvested for staining with Propidium Iodine (PI) for 30 min at 37 ^o^C. Fluorescence intensities (FL2-A channel) are presented in arbitrary units on a logarithmic scale as a measure of the amount of staining per cell. Representative photomicrographs of ECs treated without or with *t*-BHP for 2 hours observed with phase-contrast light microscope. Scale bar indicated as 80 μm. **(C)** Statistical analyses of flow cytometric data. The percentages of ECs in SubG1, G0/G1, S, and G2/M phases were represented with mean ± S.E.M. of at least three independent experiments of each group performed in triplicate. The fractions of apoptotic cell (SubG1 peak) percentages were increased gradually in *t*-BHP-treated cells. * p<0.05, ** p<0.01, and *** p<0.001, compared to Cont group.

The endothelial cells (ECs) were treated with *t*-BHP (50, 100, 250, 500, or 1000 μM) or vehicle for different times and cell viability determined by MTT assay demonstrating that *t*-BHP inhibited the growth of ECs almost in time- and dose-dependent manner (Figure 1A). At 24 h of *t*-BHP treatment all cell viability decreased significantly and higher test doses of *t*-BHP caused it decreased to below 60 % (59.0 %, 53.3 %, and 38.0 % at doses of 250, 500, and 1000 μM) of the control value. A phase contrast microscopic study demonstrated that lower test doses of *t*-BHP (50 and 100 μM) treatment for 24 h had no obvious adverse effects on cell morphology, whereas higher doses of *t*-BHP (250, 500, or 1000 μM) could diminish the cell numbers, round up the cell shapes, and reduce the cell size with condensed and vacuolated nuclei (Figure 1B). ECs treated with *t*-BHP were also stained with PI and analyzed the cell cycle by flow cytometry. Histograms indicated as the distributions of DNA contents in ECs administrating with lower doses of *t*-BHP showed no apparent influences in the G0/G1, S, and G2/M phases, as well as the proportion of apoptotic cells (subG1 phase) compared to the control cells (Figure 1C). However, when the concentrations of *t*-BHP increased, the apoptotic index in ECs elevated in a dose-dependent manner, associated with reduced G0/G1- and G2/M- and raised subG1- and S-phase cell populations. Those indicated that the induction of cell death and the retardation of cell proliferation after exposure to higher test doses of* t*-BHP.

### Effects of *t*-BHP on endothelial cell apoptosis and necrosis

**Figure 2:**
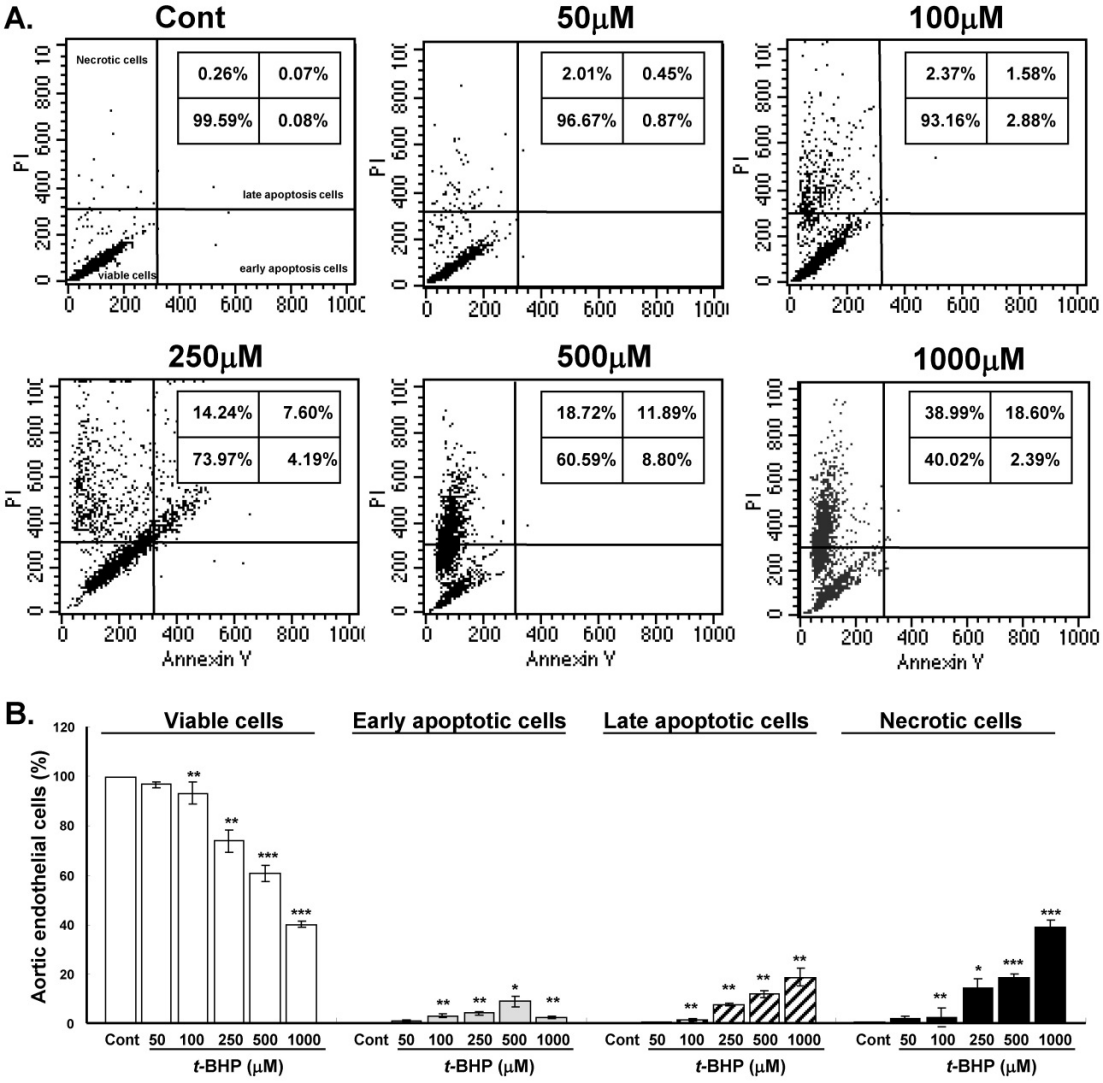
** Effects of *tert*-butyl hydroperoxide (*t*-BHP) on stimulating apoptosis and necrosis. (A)** Representative flow cytometry results of rat aortic endothelial cells (ECs). The ECs were treated without or with *t*-BHP and stained with Annexin V-FITC/PI. Schematic of results expected using this assay to ascertain cells undergoing early apoptosis (right lower quadrant), late apoptosis (right upper quadrant), and necrosis (left upper quadrant). **(B)** Quantification of the data presented in panel A with mean ± S.E.M. of at least three independent experiments. * p<0.05, ** p<0.01, and *** p<0.001, compared to Cont group.

The cytotoxic mechanism of *t*-BHP was further identified by flow cytometry double stained with Annexin V and PI (Figure 2A). Proportion of viable cells (low PI and low Annexin V staining) decreased significantly in cells treated with doses higher than 100 μM (93.2 % to 40.0 %) when compared with untreated controls (Figure 2B). Cells started to yield remarkable early apoptosis (low PI and high Annexin V staining) and late apoptosis (high PI and high Annexin V staining) when exposed to higher test doses of* t*-BHP. Significantly augmented necrotic cells (high PI and low Annexin V staining) were found in ECs treated with highest dose of *t*-BHP (39.0 % vs. 0.3 % of Cont). It indicated that higher doses of *t*-BHP not only induce apoptosis but also trigger necrotic cell death of ECs.

**Figure 3:**
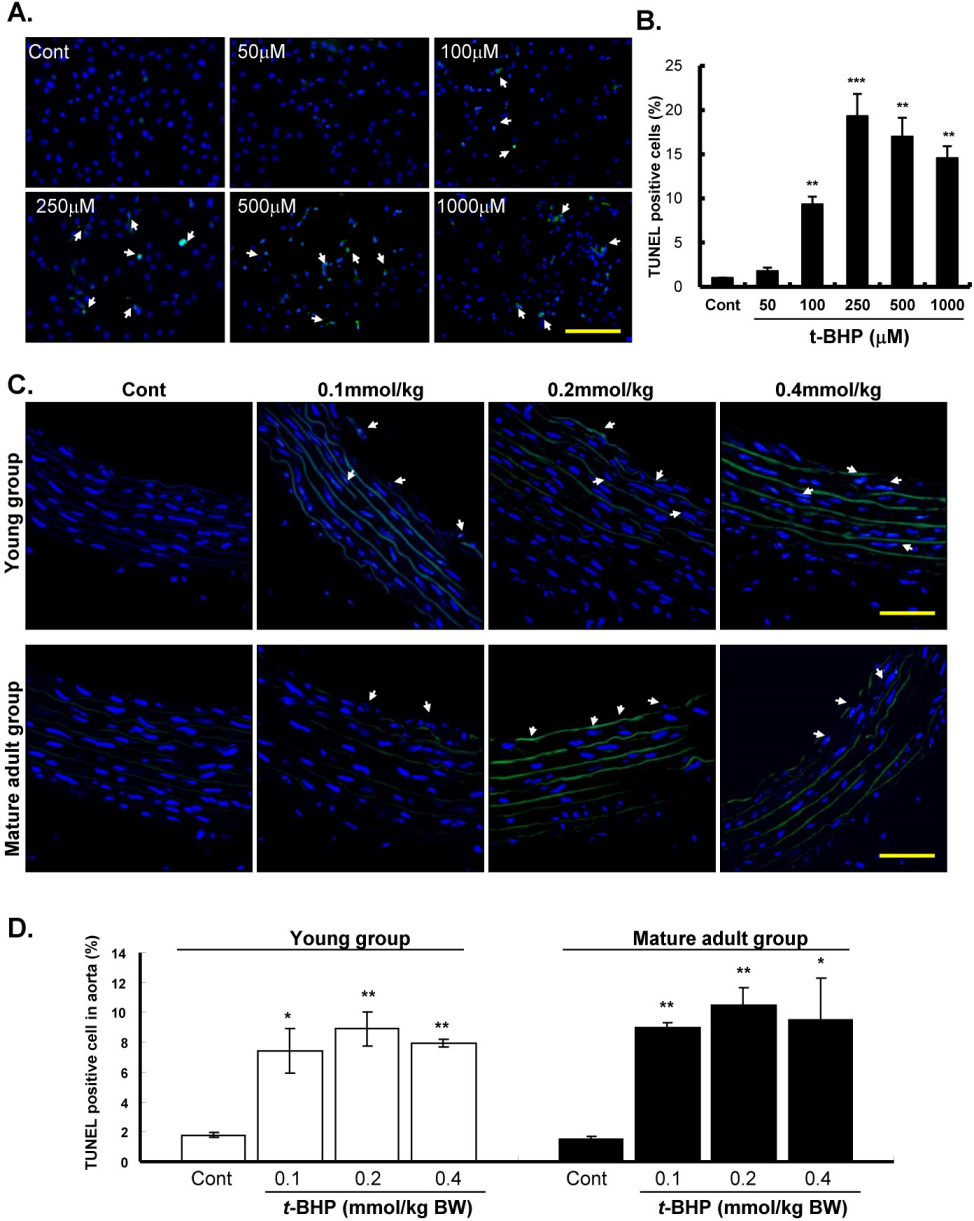
** Effects of *tert*-butyl hydroperoxide (*t*-BHP) on apoptosis.** TUNEL assay of rat aortic endothelial cells (ECs). TdT-mediated dUTP-biotin nick end represents TUNEL-positive (arrowhead), i.e. apoptotic cells. Blue color represents cell nuclei counterstained with DAPI. **(A)** *t*-BHP-treated cultured ECs contained more positive cells (green color) compared with the controls (Cont). Scale bar indicated as 80 μm. **(B)** Quantitative analysis of TUNEL-positive to DAPI-stained cell ratio. Significantly higher levels were found in cells treated with* t*-BHP at concentration higher than 100 μM in comparison with the Cont. Data were from at least three independent experiments and represented as mean ± S.E.M. * p<0.05, ** p<0.01, and *** p<0.001, compared to Cont group. **(C)** Representative sections for TUNEL assay of rat aortic rings from *t*-BHP-treated groups. Scale bar indicated as 20 μm. **(D)** Quantitative results showed more positive TUNEL index, compared with the Cont at 6-week-old (the young group) and 24-week-old (the mature adult group). Data were from 4 rats in each group and from three independent experiments. * p<0.05, and ** p<0.01, compared to Cont group.

The effects of *t*-BHP on apoptosis of ECs (Figure 3A & B) and descending aortic rings (from the young and mature adult rats) (Figure 3C & D) were further confirmed by TUNEL assay. Our results showed that TUNEL-positive nuclei were hardly observed in the Cont groups, but were prevalent when treated with *t*-BHP. Quantitative analyses showed that the proportion of apoptotic cells was tremendously higher at 250 μM (19.3 %) of *t*-BHP but declined as the highest test doses of 500 μM (17.0 %) and 1000 μM (14.6 %), depicting the necrotic effect of *t*-BHP in ECs (Figure 3B). The impacts of *t*-BHP in aortic tissues were also shown that increased percentages of positively stained cells from 1.8 % (Cont) to 8.9 % (0.2 mmol/kg) in the young group and 1.5 % (Cont) to 10.5 % (0.2 mmol/kg) in the mature adult group (Figure 3D). However, the percentages of TUNEL positive cells decreased slightly in the dose of 0.4 mmol/kg.

### Effects of *t*-BHP on capase-3 activity, apoptotic- and cell-cycle regulatory protein levels

**Figure 4:**
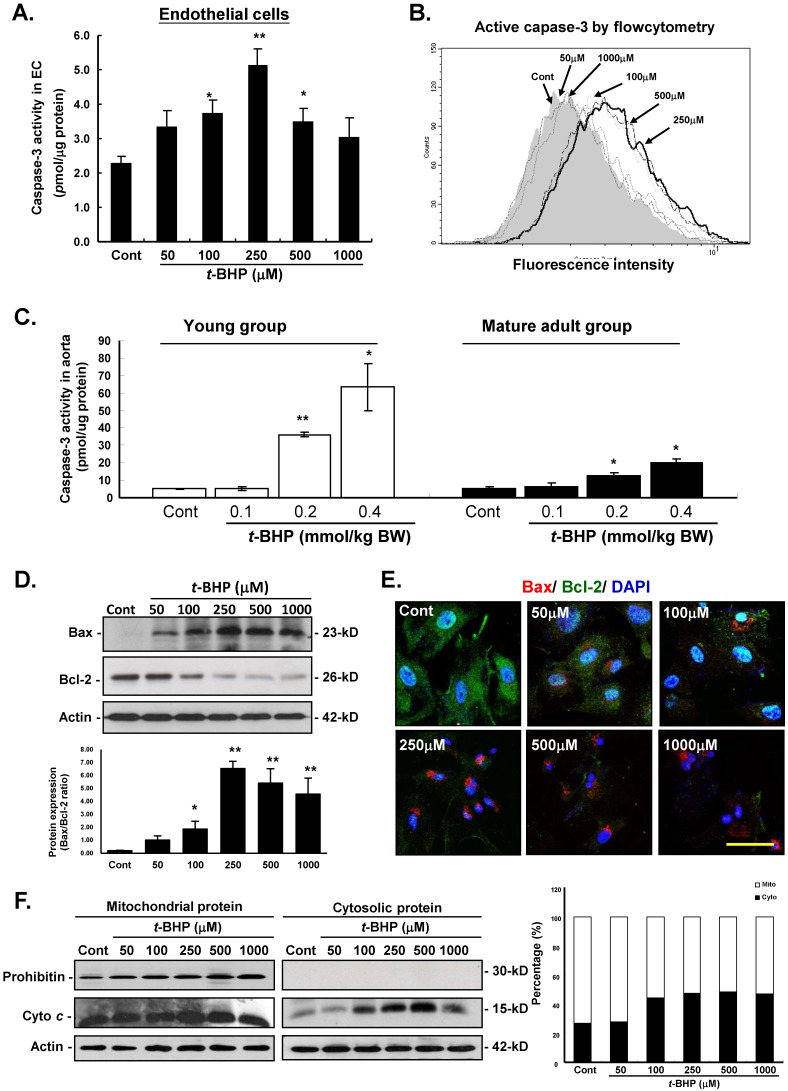
** Effects of *tert*-butyl hydroperoxide (*t*-BHP) on intrinsic pathway of apoptosis. (A)** Caspase-3 colorimetric analysis in rat aortic endothelial cells (ECs). The caspase-3 activity* t*-BHP was significantly elevated (250 μM) by 2.24-fold, compared with the Cont. **(B)** Flow cytometric study in ECs. Cells treated with *t*-BHP expressed higher caspase-3 activity (peak of caspase-3 activity deviating to the right). **(C)** Caspase-3 activities in aortic tissues. Higher doses of *t*-BHP (0.2 and 0.4 mmol/kg BW) amplified capspase-3 activities by 7.1 and 12.5 fold (the young group) and 2.4 and 3.8 fold (the mature adult group), compared with the Cont groups. Data were from six animals in each group and three independent experiments were performed in triplicate from cells. Data were represented with mean ± S.E.M. * p<0.05, and ** p<0.01. **(D)** Immunoblotting analysis of Bax and Bcl-2 proteins of the Bcl-2 family. *t*-BHP substantially up-regulates the crucial pro-apoptotic factors (Bax) and down-regulates the key anti-apoptotic proteins (Bcl-2). Quantitative results confirmed the increased Bax-to-Bcl-2 ratio in ECs treated with* t*-BHP. Representative sets of data were from three independent experiments and Actin was used as a loading control. The density of Bax/Bcl-2 ratio was converted to grayscale value and normalized to Cont. * p<0.05, and ** p<0.01. **(E)** Immunofluorescence analyses of the cellular distribution of Bcl-2 (green fluorescence) and Bax (red signals) proteins in ECs. Increased expression of Bax and decreased expression of Bcl-2 could be clearly observed in *t*-BHP-treated ECs at concentration higher than 250 μM. Blue color represents cell nuclei counterstained with DAPI. Scale bar indicated as 10 μm. **(F)** Immunoblotting study of mitochondrial and cytosolic cytochrome *c*. The ample presence of a specific mitochondrial marker, prohibitin, in the mitochondrial extracts and absence of this marker in the cytosolic fractions (upper lane) demonstrates the relative purity of both protein fractions. The abundance of cytochrome* c* was increased in the cytosol (right panel, lane 2) in *t*-BHP-treated ECs at concentration higher than 100 μM. On the right, summary data on the cellular distribution of cytochrome *c* are presented. Mito, mitochondrial fraction of cytochrome *c*; Cyto, cytosolic fraction of cytochrome *c*.

To outline the cellular mechanism responsible for the pro-apoptotic action of *t*-BHP, several decisive apoptotic regulating mediators were investigated in this study. Firstly, caspase-3 activity was examined by colorimetric (Figure 4A) and flow cytometric (Figure 4B) analyses in ECs treated with *t*-BHP. Results showed that significant up-regulation of these indicators were found at the dose higher than that of 100 μM, and peaked at 250 μM when compared with the Cont. In the animal study, results also revealed that caspase-3 activities in aorta from the *t*-BHP treated groups (the young and mature adults) were significantly activated in a dose-dependent manner, and these adverse side effects were more dominant in the young rats (Figure 4C).

The effects of the pro-apoptotic and anti-apoptotic proteins in the Bcl-2 family were further investigated by immunoblotting and immunofluorescent analyses in ECs. Our data displayed that *t*-BHP not only activated the pro-apoptotic Bax and decreased the abundance of anti-apoptotic Bcl-2 in a dose-dependent manner (Figure 4D). Figure 4E also showed the immunofluorescent results of cytosolic expressions of Bax and Bcl-2 were comparable with those of immunoblotting analyses. Moreover, immunoblotting analysis of the protein fractions from mitochondria and cytosolic further demonstrated that cytosolic fraction of cytochrome *c* (Cyto *c*) was 27.0 % of the total contents (cytosolic plus mitochondrial) in the Cont group, increased to 44.2 %, 47.6 %, 48.3 %, and 47.0 % in ECs treated with *t*-BHP (at the doses of 100, 250, 500, and 1000 μM), indicating that *t*-BHP induced redistribution of Cyto *c* from mitochondria to cytosol (Figure 4F).

### Effects of *t*-BHP on SA-β-Gal staining and telomerase activity

**Figure 5:**
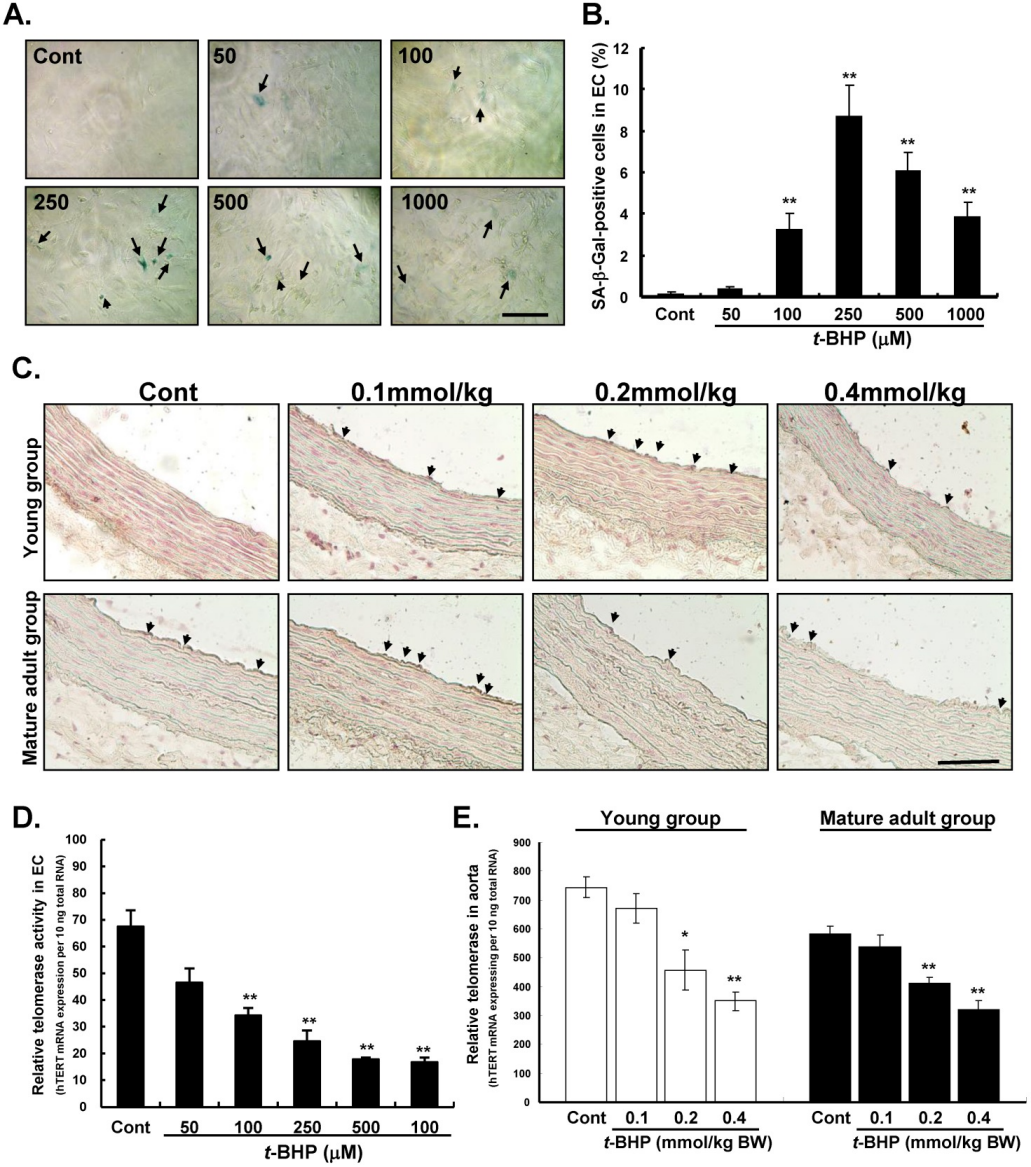
** Effects of *tert*-butyl hydroperoxide (*t*-BHP) on senescent phenotype and telomerase activity. (A)** Staining for senescence-associated β-galactosidase (SA-β-Gal) activity in endothelial cells (ECs). The* t*-BHP-treated cultured cells contained more positive cells (green color, arrowhead), compared with the Cont. Scale bar indicated as 20 μm. **(B)** Quantitative analysis of cellular senescence in ECs. The ratio of positive labeling cells was significantly higher in *t*-BHP-treated cells (concentration higher than 100 μM), compared with Cont group. **(C)** Staining for SA-β-Gal activity in rat thoracic aortas. Scale bar indicated as 50 μm. **(D) & (E)** Quantitative expression levels of telomerase activities in ECs and aortic tissues. The *t*-BHP (100 μM) remarkably inhibited telomerase activity to 41.6% in ECs, compared with the Cont. Additionally, when the dose levels greater than 250 μM in ECs conferred an additional benefit. In aorta, the dose levels of *t*-BHP higher than 0.2 mmol/kg BW soundly suppressed the telomerase activities. All data are presented as mean ± S.E.M. (n=3 in each group). Significant difference is indicated by * p<0.05 and **p<0.01, compared to Cont group.

To determine whether *t*-BHP induces premature senescence, we then examined SA-β-Gal activity in ECs and aortic tissues. When cells were treated with *t*-BHP, these morphological features of cellular senescence emerged (green color) (Figure 5A). Quantitative analyses showed that numbers of positively-SA-β-Gal stained cells increased and then declined along with the doses of *t*-BHP increased (0.4 %, 3.2 %, 8.7 %, 6.3 %, and 3.8 % at doses of 50, 100, 250, 500, and 1000 μM vs. 0.1 % of Cont, all p values <0.01 vs. Cont) (Figure 5B). The SA-β-Gal activity was also observed in thoracic aortas obtained from the young and mature adult groups of rats (Figure 5C). The SA-β-Gal-positive cells were predominately located on the luminal surface of aorta from the rat treated with higher doses of *t*-BHP (0.2 and 0.4 mmol/kg).

To further determine the role of telomerase in the senescence-stimulating action of *t*-BHP on ECs and aortic tissues, telomerase activity (hTERT mRNA expression to total mRNA) was also performed by the TRAP assay. Results showed that telomerase activity was significantly reduced in ECs treated with *t*-BHP in a dose-dependent way (34.2, 24.6, 17.7, and 16.7 at doses of 100, 250, 500, and 1000 μM vs. 67.5 of Cont, all p values <0.01 vs. Cont) (Figure 5D). Finally, we examined telomerase activity in aorta and results indicated that the effective dose of *t*-BHP to induce aortic senescence in rat was higher than 0.2 mmol/kg (Figure 5E).

### Effects of *t*-BHP on cellular adjustments to inflammatory-, apoptotic-, and cell cycle-regulating proteins

**Figure 6:**
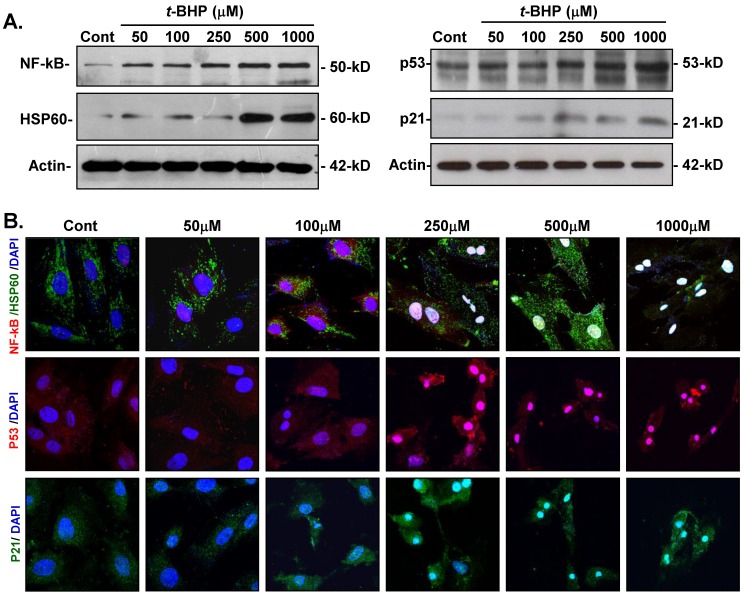
** Effects of *tert*-butyl hydroperoxide (*t*-BHP) on activation of inflammation and apoptotic signaling. (A)** Immunoblotting study of NF-kB, HSP60, p53, and p21. The expressions of NF-kB were up-regulated in endothelial cells (ECs) in response to *t*-BHP treatment, while highest dose levels of *t*-BHP (500 and 1000 μM) significantly stimulated the HSP60 expressions. p53 and p21 in cellular extracts of *t*-BHP were simultaneously increased by ECs in a dose-dependent manner. **(B)** Immunofluorescent microscopy. Cellular distribution of NF-kB (red signal), HSP60 (green fluorescence), p53 (red signal), and p21 (green fluorescence) in ECs treated with various doses of *t*-BHP. The blue signals represent ECs counterstained with DAPI. Scale bar indicated as 20 μm.

To determine whether *t*-BHP could trigger inflammatory-induced necrosis and initiate the cell cycle- and apoptosis-regulatory function in ECs, protein expressions of inflammatory-related and cell cycle-mediated proteins were investigated. Immunoblotting analysis revealed that NF-kB was obviously increased in cells treated *t*-BHP in a dose-related manner, while HSP60 expression was significantly induced by higher doses of *t*-BHP (500 and 1000 μM) (Figure 6A). Additionally, both HSP60 and NF-kB were markedly up-regulated and co-localized in *t*-BHP-treated ECs as observed by immunofluorescent analyses (Figure 6B). Abundance of p53 was also found in ECs treated with *t*-BHP in response to the dose, while p21 expression increased at higher doses and co-localized with p53.

## Discussion

*t*-BHP is typically used as a compound to underline the mechanism of cell injury initiated by acute oxidative stress not only in *in vitro* but also *in vivo* studies 12,20,22,23,26,37. In this study, we have for the first time demonstrated that *t*-BHP, a short-chain analog of lipid hydroperoxide, impedes survival of aortic endothelial cells by inducing inflammatory outcomes of apoptosis, premature senescence, and necrosis in a dose-dependent manner mediated by apoptotic- and cell cycle-regulator proteins.

### *t*-BHP induces apoptosis, necrosis, and inflammatory induced vascular dysfunction

Vascular endothelial cells play potent roles in regulating vessel integrity, immune responses, angiogenesis, and other physiological functions. Endothelial dysfunction induces several pathophysiological conditions and increase the risk of diabetes, atherosclerosis, hypertension, and other vascular diseases 1. Intima-media thickness (IMT) of the descending thoracic aorta has been reported to be correlated with the blood flow in response to coronary artery disease and stroke 6,39. A previous report indicated that isolated rat thoracic aorta treated with *t*-BHP enhanced contraction by about 140 % within a short time by disturbing the contractile response to Angiotensin II 24. Other researches also demonstrated that *t*-BHP significantly induced endothelium-dependent contraction of thoracic aorta in diabetic rats 25,40. An earlier publication indicated that mice given an intra-cerebroventricular (ICV) injection of *t*-BHP (110 mg/Kg) for 24 h caused a time-dependent increase in mortality rate and an initiation of lipid peroxidation in brain tissue 22. Our *in vivo* experiments revealed that the thoracic aortic diameter (or radius) and wall thickness (IMT) were greater in mature adult rats than in young rats. Specifically, *t*-BHP led to aortic narrowing in the young group and vascular wall thickening in the mature adult group. It is known that the narrowing of the blood vessel causes vascular resistance and increase cardiac output as well as blood pressure, which contributes to the development of early hypertension 41. Another study also proposed that increased arterial wall thickness could induce endothelial inflammation and might be associated with a greater risk of vascular dysfunction 2. Our data supported the hypothesis that an increase in the wall lumen ratio induced by *t*-BHP acted as an amplifier in hypertension in mature or elderly adult rats. More seriously, *t*-BHP exacerbated endothelial dysfunction in young rats.

Blood vessels are mainly composed of endothelial cells, which form the inner and luminal layer to support the surrounding vessel wall. Aortic endothelial cells are the most important part involved in coordinating the systemic circulation and endothelial dysfunction 1,2,41. Altered endothelial cell morphology resulting from external stress has been reported to be capable of inducing intrinsic pathway-mediated defense mechanisms to avoid cell death 42. Therefore, it is considered to be a key event involved in the development and progression of many cardiovascular diseases, including hypertension, atherosclerosis, and type II diabetes 2,3,26. Apoptosis, well known as programmed cell death or cellular suicide, can lead to activation of caspases and induce the human inflammatory diseases and cardiovascular dysfunction 43. Our study exhibited a dose-dependent proliferative and cytotoxic effect of *t*-BHP on rat aortic endothelial cells via the induction of apoptosis and senescence at relatively high starting doses (100 and 250 μM), and initiation of necrosis and inflammation at the highest tested doses (500 and 1000 μM). Similar results showed that human corneal endothelial cell treated with *t*-BHP (0-5000 μM for 2h) generated a dose-dependent cytotoxic effect, including a decrease in cell viability, change in intracellular ROS, and induction of the caspase cascade pathway 20. As we all know, *t*-BHP is able to be a ROS generation agent by inducing lipid peroxidation. The details are as follows: mouse brain ECs treated with 50 μM *t*-BHP led to more than 50 % of cell death, almost 200 % of lactate dehydrogenase (LDH) release, 3.5-fold higher in ROS production, 4-fold increase in release of Cyto *c* from the mitochondria into the cytosol, 2.5-fold higher in caspases activity, and more than 17-fold higher in *t*-BHP-induced apoptosis than in controls 20-21.

Further researchers also indicated that when rat astroglial cells were treated with 100 μM of* t*-BHP, they exhibited a notable production of cytotoxicity, such as increased apoptosis (up to 1.8 fold), augmented oxidative stress (more than 5-fold), decreased glutathione (GSH) synthesis (41 %), and diminished cytosolic expressions of cAMP-response element-binding protein (CREB) and CREB-phosphorylated (CREB-P) (27 % & 49 %, respectively), compared to untreated control cells 15. It was suggested that a higher dose of *t*-BHP (500 μM) increases higher cell death rates and induces endothelial necroptosis by activating the p38 mitogen-activated protein kinase (MAPK) signaling pathway, while a lower concentration of *t*-BHP (50 μM) induces the endothelial caspase-mediated apoptosis in human umbilical vein endothelial cells (HUVECs) 37. Moreover, cultured human trophoblast-derived cells administrated with 300 μM of *t*-BHP showed markedly increased apoptotic index (up to 3 times) and decreased migration rate (about 40 %) 19. Ali (2018) and colleagues reported that relative biological effectiveness of low dose *t*-BHP (200 μM) induces hepatotoxicity by inhibiting HepG2 cell proliferation (49 % inhibition compared to the control) 16. Another publication then indicated that *t*-BHP at the concentration of 1000 μM not only induces reduction in viability (69 % reduction) but also increases ROS formation and cspase-3 activity 12.

**Figure 7:**
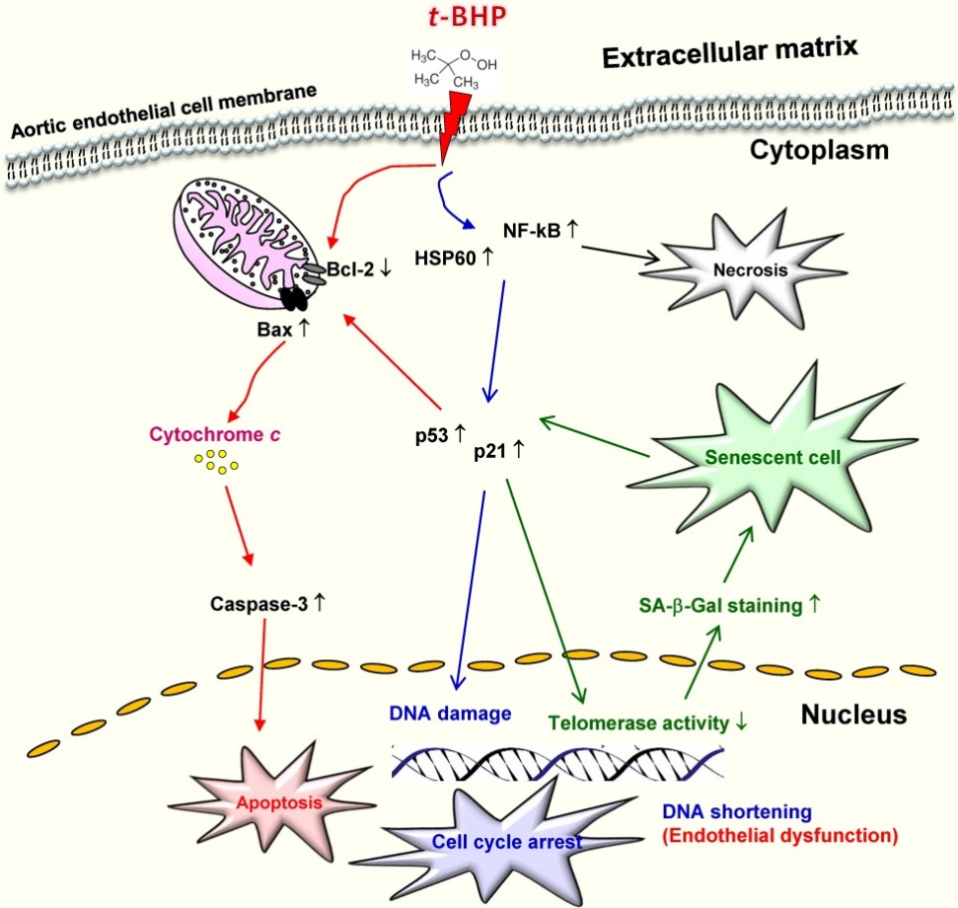
Schematic diagram showing the possible mechanism that *tert*-butyl hydroperoxide (*t*-BHP) contributed to initiation of cellular death, necrosis and cellular senescence in vascular endothelial cells.

In terms of *in vivo* research, one animal study demonstrated that time- and dose-dependent oxidative stress induced hepatotoxicity in male rats treated with *t*-BHP, and indicated that one single dose at 1 mmol/kg is suitable for evaluating the antioxidant activity of compounds of interest 33. Recent study showed that male mice treated with a single dose of *t*-BHP (2 mmol/kg) led to elevated ALT and AST levels in the liver 12. Another research also reported that *t*-BHP at a high dose can induce irreversible injury to hair-follicle growth in neonatal rats, whereas this damage by short-term administration with lower concentrations is able to be reversed by antioxidants 44. Of note, it also found mature adult rats that received 5 doses of *t*-BHP (20 μM) for 8 weeks insignificantly increased the serum levels of total cholesterol, high density lipoprotein, triglycerides, C-creative, and lipid peroxidation 45. These dose- and cell type-dependent differential properties from *in vitro* and *in vivo* studies provided evidence that *t*-BHP at high concentrations induces severe cell damage by activation of both intrinsic apoptosis pathway and inflammatory responses by ROS-mediated mitochondrial pathway, and finally increases its susceptibility to cardiovascular diseases (Figure 7).

### *t*-BHP exacerbates vascular cell senescence and inflammatory signaling to promote endothelial dysfunction

Vascular function is related to the vessel diameter in response to the endothelium's ability to defend a variety of pathophysiological stimuli 6. Our research showed that higher doses of *t*-BHP exacerbated the cardiovascular dysfunction by the induction of arterial wall thickening and initiation of cellular senescence, apoptosis, and necrosis in adult animals. Aging or premature senescence is known to be a potent risk factor in the development of cardiovascular diseases, which is attributed to the development of vascular endothelial dysfunction 1,6. A broad range of cellular changes and functional alterations usually accompanies the senescence process and apoptosis. It has been disputed that senescence and apoptosis are two independent outcomes in cells subjected to oxidative stresses, even though the same stressor is often capable of inducing either senescence or apoptosis. These two effects do share some important elements in their signaling cascades, such as being exemplified by the activation of p53 tumor suppressor and that exposure of cells to oxidative stress-causing agents can directly induce p53-medaited cell cycle arrest and apoptosis 30,46.

Senescence is considered to be a permanent growth arrest, in which cells remain metabolically active, but are fully refractory to mitogenic stimuli. However, the consequence of senescence and senescence-associated changes have been suggested to contribute to the loss of normal organ function and senescent cells are believed to survive for years under appropriate tissue culture environment to resist apoptosis 29. Stress-induced premature senescence (SIPS), carried out daily or on alternate days, is a common non-lethal treatment used to induce cells to undergo chronic oxidative stress and trigger premature aging 26,30. Our data found that high doses of *t*-BHP (100 and 250 μM) trigger cellular apoptotic and senescent phenotypes, however the higher doses (500 and 1000 μM) not only cause apoptosis but even necrosis. Similar to our results, lower doses of *t*-BHP (30 μM) for treating WI-38 fibroblast cells displayed enlarged cellular appearance, a higher frequency of SA-β-Gal staining, and decreased growth rate 27. Another study also pointed out that treatment of *t*-BHP (100 μM) resulted in apoptotic cell death. These apoptosis-inducing factors include release of Cyto *c*, activation of caspase-9 by Apaf-1, and initiation of effectors caspases, such as caspase-3 15. Intrinsic apoptosis is known to induce mitochondrial-membrane permeability through the opening of the permeability transition pore, thus allowing the release of Cyto *c* into the cytosol. Our results suggest that p53/p21 signaling pathways mediate the process of cell cycle arrest as well as premature senescence (Figure 7). It also indicated that those apoptotic-related proteins (p53, p21, and Bax) were highly upregulated in *t*-BHP-treated cells and ultimately induced vascular endothelial cell dysfunction in rats. However, further studies are needed to investigate interactions between the p53-medaited apoptotic and *t*-BHP-activated inflammatory pathways, especially at the senescence-initiation stage.

ROS plays potential role in modulating cellular signaling molecules, such as the NF-kB activity, which drives the transcription of inflammatory genes and activation of the immune pathways. This event is able to accelerate the progression of endothelial damage 6. Furthermore, increased expressions of heat shock proteins (HSPs) have been shown to be responsive to the ROS and lead to oxidative stress 43. Most importantly, HSP60 is the mitochondrial chaperonin that has been shown to interact with the pro-apoptotic protein, Bax, as well as mediate the NF-kB-dependent survival signaling in cells exposed to oxidative stress 47. It is confirmed that NF-kB activation plays a role in age-related endothelial dysfunction. On the other hand, the mitochondria was suggested to be a major source of ROS generation in aged endothelium; therefore, an activated endothelium is considered as a component of endothelial dysfunction leading the recruitment of circulating inflammatory cells 1,6. Recent studies also reported that when HepG2 cells are exposed to higher test dose of *t*-BHP (200 μM), the ROS levels increase to 100 %. In terms of male ICR mice administrated with a single dose of *t*-BHP (2 mM/kg) would elevate the serum ALT to 446.7 % and AST levels to 364.9 % compared to the control group 16. The abovementioned results indicated that *t*-BHP triggers the generation of harmful free radical intermediates to induce the production of the highly reactive hydroxyl radicals. Additionally, exposure of fatty hepatocytes to higher dose of *t*-BHP (for 60 min with 250 μM) significantly induces a 2-fold increase of malondialdehyde (MDA) production compared to lean hepatocytes 10. To sum up, *t*-BHP induces oxidative damage which is involved in the activation of pro-inflammatory transcriptional pathway and the initiation of the intrinsic cell death pathway. This occurs through the induction of the transcription of nuclear NF-kB, upregulation of caspase-3 activity, and suppression of the expressions of Bcl-2 and p38 MAPK in human fibroblast cells 48 and in human umbilical vein endothelial cells 49.

In conclusion, to the best of our knowledge, this study was the first to show that *t*-BHP exerts various growth inhibitory actions on rat aortic endothelial cells and aortic tissues stimulating apoptosis, senescence, and necrosis in dose-related diversity. Accordingly, *t*-BHP has a great impact in inducing different forms of death of endothelial cells, resulting in cardiovascular dysfunction. This study provides pathobiological evidence and suggests the dose of *t*-BHP should be lowered to avoid any adverse effects.

## Figures and Tables

**Table 1:** Effects of *tert*-butyl hydroperoxide (*t*-BHP) on body weight and morphometric parameters of thoracic aorta from young and mature adult rats.

		*t*-BHP (mmol/kg BW)		
Group	Cont	0.1	0.2	0.4
**Young** (6-week-old)				
**Body weight**				
Baseline (g)	159.6±2.0	158.9±0.9	158.5±3.4	159.2±3.3
Final (g)	253.5±3.0	252.4±4.7	238.6±4.7*	243.1±5.2
Weight Gain (%)	59.1±2.3	59.1±2.7	50.7±2.2*	52.9±2.5
**Thoracic aorta**				
Radius (mm)	2.47±0.11	2.64±0.12	2.33±0.07	2.38±0.09
Intima-medial thickness (μm)	174.2±5.4	175.5±12.8	146.0±3.1*	149.4±1.4*
			
**Mature adult** (24-week-old)				
**Body weight**				
Baseline (g)	471.5±4.5	470.7±4.3	470.8±2.5	473.0±2.8
Final (g)	557.3±4.0	576.7±6.1	563.5±10.6	534.6±6.1*
Weight Gain (%)	18.2±0.7	22.6±1.7	19.7±2.3	13.1±1.4*
**Thoracic aorta**				
Radius (mm)	2.87±0.05	2.73±0.08	2.71±0.01	2.96±0.06
Intima-medial thickness (μm)	189.3±0.5	199.3±0.5**	200.0±2.4*	202.7±2.5*

Representative values are shown as mean ± S.E.M., (Young group, n=12; Mature adult group, n=6); *p<0.05 and **p<0.01, compared to the Cont group.

## References

[1] Sena CM, Pereira AM, Seiça R (2013). Endothelial dysfunction - A major mediator of diabetic vascular disease. Biochimica et Biophysica Acta.

[2] Su JB (2015). Vascular endothelial dysfunction and pharmacological treatment. World J Cardiol.

[3] Buja LM, Ottaviani G, Mitchell RN (2019). Pathobiology of cardiovascular diseases: an update. Cardiovascular Pathology.

[4] Corti R, Fuster V, Badimon JJ (2003). Pathogenetic concepts of acute coronary syndromes. J Am Coll Cardiol.

[5] Erbel R, Eggebrecht H (2006). Aortic dimensions and the risk of dissection. Heart.

[6] Edirisinghe I, Burton-Freeman BM (2014). Age associated endothelial dysfunction: Role of oxidative stress, inflammation and Western Diet. Nutr Aging.

[7] Defagó MD, Elorriaga N, Irazola VE (2014). Influence of Food Patterns on Endothelial Biomarkers: A Systematic Review. J Clin Hypertens.

[8] Jackson SJ, Andrews N, Ball D (2017). Does age matter? The impact of rodent age on study outcomes. Lab Anim.

[9] Kawamura H, Tanaka S, Ota Y (2018). Dietary intake of inorganic phosphorus has a stronger influence on vascular-endothelium function than organic phosphorus. J Clin Biochem Nutr.

[10] Kučera O, Endlicher R, Roušar T (2014). The effect of *tert*-butyl hydroperoxide-induced oxidative stress on lean and steatotic rat hepatocytes in vitro. Oxid Med Cell Longev.

[11] Viji P, Venkateshwarlu G, Ravishankar CN (2017). Role of plant extracts as natural additives in fish and fish products - a rReview. Fish Technol.

[12] Jin SW, Hwang YP, Choi CY (2017). Protective effect of rutaecarpine against t-BHP-induced hepatotoxicity by upregulating antioxidant enzymes via the CaMKII-Akt and Nrf2/ARE pathways. Food Chem Toxicol.

[13] Caprioli G, Maggi F, Bendif H (2018). Thymus lanceolatus ethanolic extract protects human cells from t-BHP induced oxidative damage. Food Funct.

[14] Gazdag Z, Máté G, Čertik M (2014). tert-Butyl hydroperoxide-induced differing plasma membrane and oxidative stress processes in yeast strains BY4741 and erg5Delta. J Basic Microbiol.

[15] Kim SC, Lee JR, Park SJ (2014). Role of 6-shogaol in tert -butyl hydroperoxide-induced apoptosis of HepG2 cells. Pharmacology.

[16] Ali MY, Jannat S, Jung HA (2018). Hepatoprotective effect of Cassia obtusifolia seed extract and constituents against oxidative damage induced by tert-butyl hydroperoxide in human hepatic HepG2 cells. J Food Biochem.

[17] Liang F, Fang Y, Cao W (2018). Attenuation of *tert*-Butyl Hydroperoxide (*t*-BHP)-Induced Oxidative Damage in HepG2 Cells by Tangeretin: Relevance of the Nrf2-ARE and MAPK Signaling Pathways. J Agric Food Chem.

[18] Holownia A, Mroz RM, Wielgat P (2009). Propofol protects rat astroglial cells against *tert*-butyl hydroperoxide-induced cytotoxicity; the effect on histone and cAMP-response-element-binding protein (CREB) signalling. J Physiol Pharmacol.

[19] Carletti JV, Correia-Branco A, Silva CR (2018). The effect of oxidative stress induced by tert-butylhydroperoxide under distinct folic acid conditions: An in vitro study using cultured human trophoblast-derived cells. Reprod Toxicol.

[20] Shin YJ, Kim JH, Seo JM (2009). Protective effect of clusterin on oxidative stress-induced cell death of human corneal endothelial cells. Mol Vis.

[21] Liu H, Mao P, Wang J (2016). Azilsartan, an angiotensin II type 1 receptor blocker, attenuates tert-butyl hydroperoxide-induced endothelial cell injury through inhibition of mitochondrial dysfunction and anti-inflammatory activity. Neurochem Int.

[22] Ko KM, Lam BYH, Schisandrin B (2002). Protects against tert-butylhydroperoxide induced cerebral toxicity by enhancing glutathione antioxidant status in mouse brain. Mol Cell Biochem.

[23] Correia S, Vaz CV, Silva AMS (2017). Regucalcin counteracts tert-butyl hydroperoxide and cadmium-induced oxidative stress in rat testis. J Appl Toxicol.

[24] Patel RJ, Patel PD, Patel MM (2009). Mechanisms of potentiation of Angiotensin II-induced contractile response of isolated rat aorta by hydrogen peroxide and tert-butyryl hydroperoxide. Indian J Pharmacol.

[25] Saini AK, Patel RJ, Sharma SS (2006). Edaravone attenuates hydroxyl radical stress and augmented angiotensin II response in diabetic rats. Pharmacol Res.

[26] Goligorsky MS, Hirschi K (2016). Stress-Induced Premature Senescence of Endothelial and Endothelial Progenitor Cells. Adv Pharmacol.

[27] Li YB, Zhong ZF, Chen MW (2013). Bisdemethoxycurcumin increases Sirt1 to antagonize t -BHP-induced premature senescence in WI38 fibroblast cells. Evid Based Complement Alternat Med.

[28] Colitz CMH, Whittington A, Carter R (2004). The effects of oxidative stress on telomerase activity and other stress-related proteins in lens epithelial cells. Exp Eye Res.

[29] Childs BG, Baker DJ, Kirkland JL (2014). Senescence and apoptosis: dueling or complementary cell fates?. EMBO Rep.

[30] Ott C, Jung T, Grune T (2018). SIPS as a model to study age-related changes in proteolysis and aggregate formation. Mech Ageing Dev.

[31] Wanga Y, Chena L, Hagiwara N (2010). Regulation of heat shock protein 60 and 72 expression in the failing heart. J Mol Cell Cardiol.

[32] Tani M, Suganuma Y, Hasegawa H (1997). Decrease in ischemic tolerance with aging in isolated perfused Fischer 344 rat hearts: Relation to increases in intracellular Na+ after ischemia. J Mol Cell Cardiol.

[33] Oh JM, Jung YS, Jeon BS (2012). Evaluation of hepatotoxicity and oxidative stress in rats treated with tert-butyl hydroperoxide. Food Chem Toxicol.

[34] EUROPA: Risk Assessment: tertiary butyl hydroperoxide (TBHP).

[35] Yeh YC, Hwang GY, Liu IP (2002). Identification and expression of scavenger receptor SR-BI in endothelial cells and smooth muscle cells of rat aorta in vitro and in vivo. Atherosclerosis.

[36] Zanetta L, Marcus SG, Vasile J (2000). Expression of von Willebrand factor, an endothelial cell marker, is up- regulated by angiogenesis factors: A potential method for objective assessment of tumor angiogenesis. International Journal of Cancer.

[37] Zhao W, Feng H, Sun W (2017). Tert-butyl hydroperoxide (t-BHP) induced apoptosis and necroptosis in endothelial cells: Roles of NOX4 and mitochondrion. Redox Biol.

[38] Yeh YC, Liu TJ, Lai HC (2015). Shikonin induces apoptosis, necrosis, and premature senescence of human A549 Lung cancer cells through upregulation of p53 expression. Evidence-Based Complementary and Alternative Medicine.

[39] Petrini J, Yousry M, Eriksson P (2016). Intima-media thickness of the descending aorta in patients with bicuspid aortic valve. Int J Cardiol Heart Vasc.

[40] Awe SO, Tsakadze NL, D'Souza SE (2003). tert-Butyl hydroperoxide-mediated vascular responses in DOCA-salt hypertensive rats. Vascular Pharmacology.

[41] Heagerty AM, Heerkens EH, Izzard AS (2010). Small artery structure and function in hypertension. J Cell Mol Med.

[42] Ma W, Zhu X, Ding X (2015). Protective effects of SS31 on t-BHP induced oxidative damage in 661W cells. Mol Med Rep.

[43] Ikwegbue PC, Masamba P, Oyinloye BE (2018). Roles of heat shock proteins in apoptosis, oxidative stress, human inflammatory diseases, and cancer. Pharmaceuticals (Basel).

[44] Wikramanayake TC, Simon J, Mauro LM (2011). tert-butyl hydroperoxide, an organic peroxide, causes temporary delay in hair growth in a neonatal rat model. Clin Exp Dermatol.

[45] Francisco NM, Aboua YG, Brooks NL (2011). Can *tertiary*-butyl hydroperoxide cause cardivascular disease?. Med Technol SA.

[46] Ayala A, F.Muñoz M, Argüelles S (2014). Lipid Peroxidation: Production, Metabolism, and Signaling Mechanisms of Malondialdehyde and 4-Hydroxy-2-Nonena. Oxid Med Cell Longev.

[47] Chun JN, Choi B, Lee KW (2010). Cytosolic Hsp60 Is Involved in the NF-kB-Dependent Survival of Cancer Cells via IKK Regulation. PLoS ONE.

[48] Bae SJ, Lee JS, Kim JM (2010). 5-Hydroxytrytophan inhibits tert-butylhydroperoxide (t-BHP)-induced oxidative damage via the suppression of reactive species (RS) and nuclear factor-kappaB (NF-kappaB) activation on human fibroblast. J Agric Food Chem.

[49] Wang L, Tang L, Wang Y (2016). Exendin-4 protects HUVECs from t-BHP-induced apoptosis via PI3K/Akt-Bcl-2-caspase-3 signaling. Endocr Res.

